# An Overview on Image-Based and Scanner-Based 3D Modeling Technologies

**DOI:** 10.3390/s23020596

**Published:** 2023-01-04

**Authors:** Styliani Verykokou, Charalabos Ioannidis

**Affiliations:** Laboratory of Photogrammetry, School of Rural, Surveying and Geoinformatics Engineering, National Technical University of Athens, 9 Iroon Polytechniou Str., 15780 Athens, Greece

**Keywords:** 3D modeling, structure from motion, multi-view stereo, segmentation, scanners, laser scanners, CT, CBCT, MRI, CMM

## Abstract

Advances in the scientific fields of photogrammetry and computer vision have led to the development of automated multi-image methods that solve the problem of 3D reconstruction. Simultaneously, 3D scanners have become a common source of data acquisition for 3D modeling of real objects/scenes/human bodies. This article presents a comprehensive overview of different 3D modeling technologies that may be used to generate 3D reconstructions of outer or inner surfaces of different kinds of targets. In this context, it covers the topics of 3D modeling using images via different methods, it provides a detailed classification of 3D scanners by additionally presenting the basic operating principles of each type of scanner, and it discusses the problem of generating 3D models from scans. Finally, it outlines some applications of 3D modeling, beyond well-established topographic ones.

## 1. Introduction

3D reconstruction of objects and scenes or even humans or parts of their bodies, tissues, and organs is a prerequisite for a great number of applications. Topographic applications of 3D reconstruction have become very common for many years, ranging from 3D city modeling to 3D reconstruction of sites, buildings, and objects for a great variety of applications, such as geometric documentation, inspection, navigation, visualization, and object identification. Except for well-established 3D modeling applications, the problem of 3D reconstruction is also posed by different, more “specialized” applications, e.g., in the fields of medicine/dentistry, computer graphics, cultural heritage, safety, search and rescue, and manufacturing. 3D models may be generated using a variety of different technologies, i.e., either using images from camera sensors or data from scanners of different categories, or even a combination of both. In this context, the aim of this article is the presentation of popular 3D modeling technologies using data from camera sensors or scanners. It aims to provide a comprehensive overview of different technologies that may be used to generate 3D models of outer or inner surfaces of different kinds of targets (objects/scenes/human (or animal) bodies).

The article starts with a theoretical presentation of the problem of 3D modeling using images in Section 2. In particular, different forms of representation of the 3D scene geometry depicted in overlapping images are described, followed by a presentation of the stages of automatic multi-image reconstruction, which is the most usual process for generating 3D models. Afterward, other methods of 3D modeling using images are briefly outlined: two-image reconstruction, conventional photogrammetric procedure, shading-based shape recovery, use of stereo-camera, and use of satellite images. Then, in Section 3, the problem of 3D modeling using scanners is discussed. This section starts with a detailed classification of 3D scanners, and a presentation of the basic operating principles of both contact scanners (laser scanners, sonar systems, radar systems, computed tomography scanners, and magnetic resonance imaging scanners) and non-contact scanners (ultrasonic scanners, coordinate measuring machines, and destructive inspection scanners) is made. Furthermore, the problem of converting different kinds of scans to 3D models is discussed in Section 3. Overall, the 3D modeling methods outlined in Table 1 are presented in this article. Finally, indicative 3D modeling applications, beyond conventional topographic ones, are discussed in Section 4, which is followed by the conclusions of this research.

## 2. Image-Based 3D Modeling

**Figure 1:**
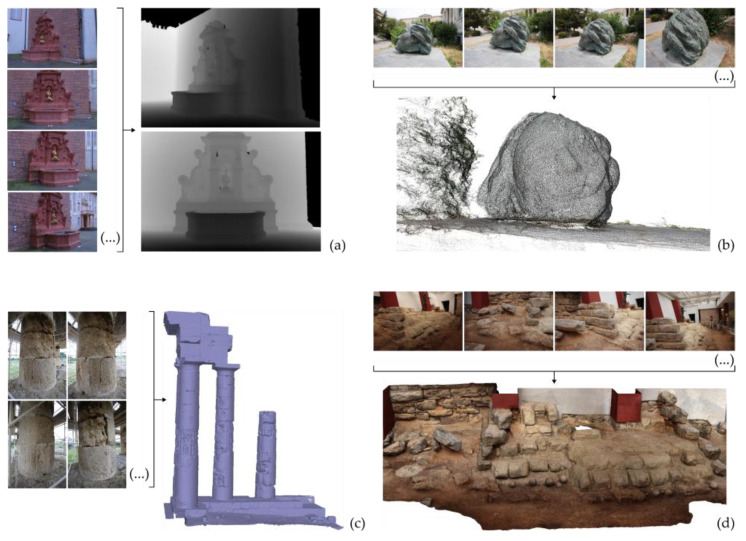
Forms of representation of 3D geometry. (**a**) Depth maps [source of images and depth maps: https://documents.epfl.ch/groups/c/cv/cvlab-unit/www/data/multiview/denseMVS.html, Accessed on 29 November 2022] [3]; (**b**) dense point cloud; (**c**) triangular mesh; (**d**) textured triangular mesh.

The reconstruction of the geometry of 3D space from images is a problem that has been tackled by the photogrammetric community since the end of the last century. Advances in the scientific fields of photogrammetry and computer vision have led to the development of automated multi-image methods that solve the problem of 3D reconstruction. Common forms of representation of the 3D geometry of the scene depicted in a set of overlapping images are listed below (Figure 1) [1,2].

- Depth map or depth maps. A depth map is a 2D representation of an image, which includes for each pixel its depth, i.e., the value of its distance from the point of capture (projection center). It is visualized as an image by converting the depth values into intensity values.
- Dense point cloud. It is a set of points in space with known 3D coordinates in a defined reference system.
- 3D model. The most common form of representation of the 3D geometry is a polygonal model (polygon mesh) consisting of a set of vertices, edges, and polygons (usually triangles, but may also be quadrilaterals or, rarely, polygons with more than four vertices), which describe the 3D surface of the scene. Texture mapping to the model is also common. While a polygon mesh represents the surface of an object, a polyhedral mesh represents (in addition to the surface) the volume occupied by an object, e.g., tetrahedral model and parallelepiped model. The voxels are structural elements of parallelepipeds, representing the smallest cube-shaped distinct part of a volume, constituting a cell of a 3D grid.

### 2.1. Multi-View Stereo

**Figure 2:**
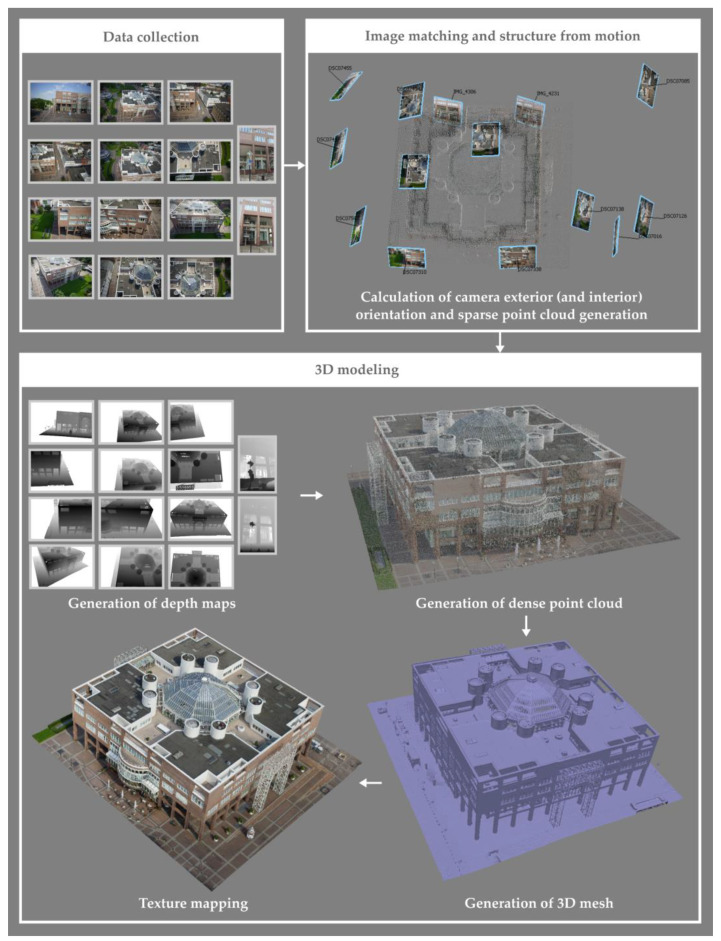
SfM-MVS-based 3D modeling. In the figure, for graphical reasons, a subset of the original images is depicted. The white areas in the depth maps correspond to parts of the images that are not used for reconstruction. Source of images used for 3D reconstruction: ISPRS/EuroSDR Benchmark for Multi-Platform Photogrammetry [33].

In the international literature, the multi-view 3D reconstruction process is usually referred to as multi-view stereo (MVS) [2]. This term is attributed to a wide range of photogrammetric and computer vision techniques, which generate different forms of representation of 3D geometry. The stages of a multi-image 3D reconstruction process are graphically illustrated in Figure 2 and are summarized below.

1. Data collection stage. It includes capturing of overlapping images (terrestrial images and/or aerial images from a manned or unmanned aircraft) and topographical measurements, if required.
2. Image orientation stage. It concerns the calculation of the exterior orientation and (optionally) interior orientation of the images and the generation of a sparse point cloud, which consists of the 3D coordinates of the tie points, i.e., homologous feature points. It is usually performed by applying methods of (a) detection of overlapping images, (b) image matching and feature tracking, and (c) structure from motion (SfM). SfM methods are mainly categorized into three categories: incremental, global, and hierarchical SfM [4,5,6]. Incremental SfM methods are the most commonly used ones. These methods introduce images incrementally into the orientation and sparse reconstruction process, as they orient one image at each iteration (e.g., [7,8,9,10,11,12]). Global SfM methods simultaneously calculate the 3D coordinates of a sparse point cloud and the exterior orientation of the images in a single bundle adjustment solution through a factorization method or a motion averaging method (e.g., [13,14,15,16,17,18]). Hierarchical SfM methods divide the problem of orientation and sparse reconstruction into smaller subproblems, which are combined in a hierarchical manner (e.g., [19,20,21,22]). Whether the sparse point cloud is used in the subsequent steps of the 3D reconstruction depends on the applied method. However, the combination of image matching and SfM methods is followed by all 3D reconstruction algorithms.
3. Depth map generation stage. It is performed via dense image matching for a subset of the overlapping image pairs of known interior and exterior orientation (or for all overlapping image pairs) and produces a set of depth maps for the reference images. Dense image matching methods can be distinguished into two main categories: local methods and global methods [23,24,25]. In local methods, the calculation of the disparity of each pixel of the reference image depends solely on the intensity values within a specified window. They follow the simplest way of producing the disparity map, as, for each pixel, they select the disparity corresponding to the largest or smallest (depending on the selected similarity measure) aggregated matching cost. In local methods, the problem of computing the disparity map is related to the minimization of a global energy function, usually defined for all pixels of the reference image, introducing, additionally, a disparity smoothness constraint for the entire image. Moreover, combinations of the above categories of methods have been introduced, such as the semi-global matching method [26] and its variants, which aim to reduce the computational complexity of global methods. Dense image matching is not applied by all MVS algorithms.
4. Dense point cloud generation stage. It is usually performed by applying some method of merging the depth maps created in stage 3 (e.g., [27,28]), or by applying some method of densification of the sparse point cloud created in stage 2 (e.g., [29]). This step is not followed by all 3D reconstruction algorithms.
5. 3D surface generation stage. It concerns the production of a polygonal (usually triangular) mesh model. Several methods have been developed for producing a 3D surface from a point cloud (derived by stage 4) [30]. Some indicative 3D meshing methods are the following: (a) methods based on Delaunay triangulation, that is, on the construction of a graph that connects points to each other, forming triangles (in 2D space) or tetrahedrons (in 3D space) with circumcircles that do not contain any points in their interior, (b) methods based on the Voronoi diagram, which is the dual graph of Delaunay triangulation, that creates a region for each point consisting of all the points that are closer to that point than to any other point, (c) methods based on the convex hull, i.e., the smallest convex polygon (in 2D space) or polyhedron (in 3D space) that includes all points of the cloud, having some of them as vertices, and (d) methods based on alpha shapes (a-shapes), i.e., a family of lines connected to the shape defined by a set of points, being a generalization of the convex hull. The above methods produce a triangular model using all or most of the points, which have to be additionally accompanied by normal vectors. Another commonly used method for reconstructing the 3D scene geometry from a point cloud is the Poisson reconstruction method [31]. If the point cloud is not accompanied by normal vectors, these can be calculated, e.g., through adjustment of a local plane at each point. In addition, in the case of noisy point clouds, the resulting surface often needs further processing. However, 3D surface generation is not solely based on a 3D dense point cloud. It can also be performed using the depth maps (stage 3), without converting them into a 3D point cloud. The process of creating a 3D surface based on depth maps allows the use of all the information contained in the original images and does not rely on the, often filtered and merged, point cloud. It is also faster, considering that it skips the step of merging the depth maps into a single point cloud.
6. Texture mapping stage. This stage, which is usually the last one of a multi-image 3D reconstruction process, generates texture and maps it to the 3D model using the images of known interior and exterior orientation. The usual procedure for generating a texture map involves projecting each polygon of the mesh to one or more images, in which it is visible, and finding the optimal image(s) for rendering texture to each polygon [32]. An indicative process for creating a texture map, making the assumptions of triangular mesh and selection of texture for each triangle from a single image, is outlined in the following: (a) projection of each triangle in the images in which it should be visible (regardless of whether it is occluded) and (b) selection of the optimal image for texture mapping to each triangle, based on various criteria, e.g., occlusions, resolution of the part of the image in which each triangle is projected, viewing angle, and relevance to neighboring pixels. The simplest method of creating the texture map is to incrementally store (“copy”) connected parts of the same image that are used to texture the triangles of the mesh. Finally, each vertex of each triangle is assigned texture coordinates from the texture map, corresponding to row and column numbers of the 2D texture map.

### 2.2. Two-Image Reconstruction

In addition to the aforementioned multi-image reconstruction process, which is the most common method of creating 3D models using images, a 3D photogrammetric product can also be derived using two overlapping images (stereo-pair). In the case of availability of only two overlapping images of known interior orientation, a variation of the procedure, presented in Section 2.1, may be followed, omitting some steps. An automated method could consist of the steps listed below.

1. Feature extraction and image matching.
2. Calculation of the relative orientation parameters of the stereo-pair (using at least five correspondences).
3. Absolute orientation of the stereo-pair (using at least three ground control points, if available and if the final 3D model is intended to be georeferenced).
4. Generation of depth map.
5. Generation of dense point cloud.
6. Generation of 3D mesh.
7. Texture mapping on the 3D mesh.

The relative and absolute orientation processes could also be replaced by a photogrammetric resection solution, performed separately for each image of the pair. In this case, steps (1–3) could be replaced by solving two separate photogrammetric resections—one for each image of the pair—using at least three ground control points.

### 2.3. Conventional Photogrammetric Procedure

Before the development and widespread use of automated feature extraction, image matching, and dense image matching algorithms, the process of 3D modeling using images was performed on digital photogrammetric stations via conventional photogrammetry processes. Such a classic process of generating a 3D product in a digital photogrammetric station is outlined below. In the case of availability of a set of overlapping images, the calculation of their exterior orientation—and in the case of uncalibrated cameras, their interior orientation too—is carried out at a digital photogrammetric station via a phototriangulation process (aerial triangulation in the case of aerial images). Tie points, before the introduction of automations in digital photogrammetric stations, were manually detected by the operator. However, modern photogrammetric stations provide the possibility of automatic extraction of tie points and image matching. In the case of using only two overlapping images taken by a calibrated camera, the orientation procedure may be performed through relative and absolute orientation calculation procedures, as mentioned in Section 2.2.

After the computation of the relative orientation (or the absolute orientation in the case of availability of ground control points) for the block of images or for the pair (in the case of availability of only two images), the process of manual stereo-plotting can be applied for production of 3D vector drawings by the operator, who wears special stereoscopic glasses that provide him/her with the possibility of stereoscopic vision in each stereo-model. One such type of glasses is liquid crystal shutter glasses, which are synchronized with the computer graphics system and work by forming slightly different images in each eye, creating a depth perception in the operator’s brain. The screen displays successively with very fast switching (over 120 Hz), in the left and right eye, the corresponding images so that the operator’s brain perceives continuous observation, having the possibility of stereoscopic vision [34]. There are also passive polarization systems, consisting of a combination of a liquid crystal screen (which adapts to the computer screen) and polarized glasses, in which each eye sees only one image [35]. Another type of glasses (which does not require special equipment) is color-coded anaglyph glasses for stereoscopic viewing using anaglyph images. The latter consist of two differently filtered color images, one for each eye. The sense of stereoscopic observation is achieved when the operator observes the images wearing the anaglyph glasses, which are of a different color for each eye, corresponding to the filter of the image projected on each eye. Today, the most common color combination is red and cyan [36].

### 2.4. Shading-Based Shape Recovery

Unlike multi-view reconstruction and two-image reconstruction algorithms that use overlapping images taken from different viewpoints to obtain the 3D geometry of the imaged scene, reflectance-based shape recovery of non-planar surfaces from one or more irradiance images is another category of methods, which aims to reconstruct the 3D shape of objects from their irradiances by using their reflection properties [37]. The class of methods that use two or more images for shading-based 3D shape recovery is referred to as photometric stereo [38]. The idea of photometric stereo is to vary the direction of incident illumination between successive images, while holding the viewing direction constant. This category of methods aims to recover the surface normals of a 3D object from various shading cues. Traditional photometric stereo methods deal with Lambertian surfaces, with perfectly diffuse reflection. However, photometric stereo methods that deal with non-Lambertian surfaces have also been introduced, either by adopting reflectance models to approximate the non-Lambertian surface properties of objects or based on deep learning [39,40,41]. In the case of usage of a single irradiance image, the problem is called shape from shading [42].

### 2.5. Usage of a Stereo-Camera

In addition to single-lens cameras and multi-camera systems, in which the 3D modeling process may follow the automatic multi-image reconstruction pipeline mentioned in Section 2.1, stereo-cameras may also be used for 3D modeling purposes. A stereo-camera is a type of camera with two or more lenses and a separate image sensor for each lens, which takes images for each sensor simultaneously and may produce depth maps, point clouds, and 3D models through photogrammetric processing [43].

### 2.6. Usage of Satellite Imagery

While the aforementioned procedures (Section 2.1, Section 2.2, Section 2.3, Section 2.4 and Section 2.5) refer to terrestrial or aerial images, a 3D modeling process using satellite imagery follows similar logic, with different methods for their orientation (georeferencing). Specifically, the orientation of satellite images can be carried out by applying an approximate or strict mathematical model [44]. In the context of approximate mathematical models (non-rigorous models), the transformation between image space and object space is expressed by generalized relations without any modeling of the physical imaging process. In the context of rigorous mathematical models, a complex mathematical model is used, with knowledge of the geometry of the receiver, which represents the physical arrangement of capturing the satellite images. After the orientation of the satellite images, the process of producing a 3D model/digital surface model may be achieved through the processes of dense image matching to generate depth map(s), dense point cloud(s), and 3D meshing.

### 2.7. Discussion

Photogrammetry-based 3D reconstruction using images includes a variety of methods that may be applied depending on multiple factors, such as the kind of images (terrestrial/aerial images from a manned or unmanned aircraft/satellite images), their number (multi-image process/two-view reconstruction/single-image reconstruction), characteristics of the image capturing process (e.g., percentage of overlap, ordered/unordered image sequences), the degree of automation of the process (fully automatic/semi-automatic/manual process), the characteristics of the object to be reconstructed, the desired form of representation of the 3D geometry, the target accuracy, etc. Nowadays, a great variety of software solutions have been made available to the public, both commercial and free ones, addressing not only photogrammetrists, but also other scientists as well, both for professional and non-professional use. The highest percentage of these software solutions belongs to the category of SfM-MVS software, as the usage of multiple images for automated 3D reconstruction is very common. Such solutions offer automated tools that solve the problem of image-based 3D reconstruction, providing user-friendly interfaces that make them an attractive solution for non-photogrammetrists as well. However, basic knowledge of photogrammetry/computer vision concepts is necessary for supervision of the whole process, including understanding any failures of the automated steps and making the necessary corrections in the parameterization or the process (e.g., deleting unnecessary/low-quality images, masking parts of images with undesirable characteristics, removing wrongly reconstructed points from the sparse point cloud, applying manual/semi-automatic point cloud and mesh processing, etc.).

## 3. Scanner-Based 3D Modeling

In this section, a taxonomy of 3D scanners is provided, the basic operating principles of each category of scanners are presented, and the problem of converting scans to 3D models is addressed, followed by a brief discussion concerning scanner-based 3D modeling.

### 3.1. A Taxonomy of Scanners

**Figure 3:**
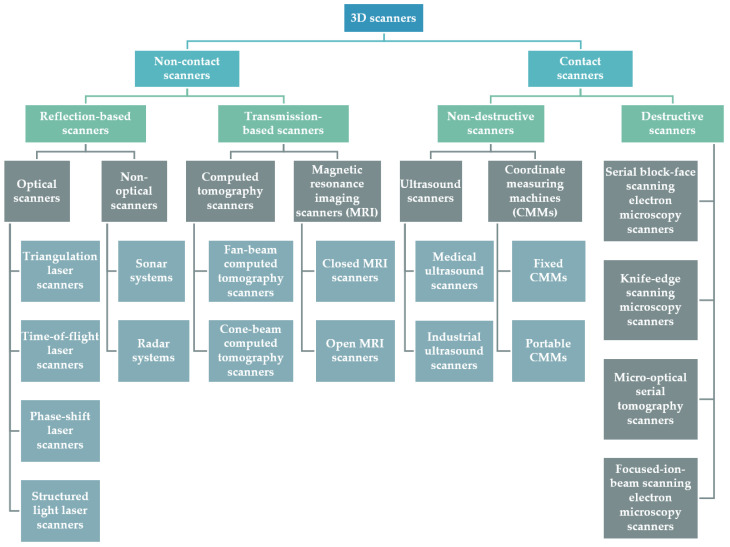
A taxonomy of 3D scanners.

Various classifications of 3D scanners have been proposed in the scientific literature [45,46,47,48,49]. Figure 3 shows a classification of 3D scanners, which is adopted in the context of this article. According to this classification, scanners may be distinguished into two main categories, as described below, depending on whether they scan the object of interest without physical contact (non-contact scanners) or whether they require physical contact to scan it (contact scanners).

1. Non-contact scanners. They scan the object without touching it. They may be further distinguished into two main subcategories: scanners based on the reflection of waves from the object being scanned (reflection-based scanners) and scanners based on the transmission of rays in the material being scanned (transmission-based) scanners.
  - Reflection-based scanners. They produce a 3D representation of the external surface of the object being scanned. Optical scanners and non-optical scanners belong to this category. Optical scanners rely on the reflection of optical radiation. These are, basically, laser scanners, and their basic principles are mentioned in Section 3.1.1. Non-optical scanners include sonar and radar systems, which are presented in Section 3.1.2.
  - Transmission-based scanners. Scanners of this type produce a 3D representation of the internal surface of the target being scanned. Computed tomography scanners and magnetic resonance imaging scanners, which are mainly used for medical purposes, belong to this category. Computed tomography scanners emit high-energy X-rays and measure the amount of radiation that passes through the object/patient being scanned. The basic principles of operation of this type of scanner are summarized in Section 3.1.3. Magnetic resonance imaging scanners use a strong magnetic field of waves and radio frequencies to create a 3D representation of the target. Their basic principles are presented in Section 3.1.4.
2. Contact scanners. They touch the surface of the object in order to scan it, producing 3D models of targets through physical contact with them. They may be further distinguished into two main categories, depending on whether or not they cause any damage/alteration/destruction of the object being scanned, as described below [50].
  - Non-destructive scanners. They do not cause any damage/alteration/destruction to the object being scanned. This category includes 3D ultrasound scanners, which touch the target (patient body in medical ultrasound scanners or other material in industrial ultrasound scanners) for the 3D representation of its internal parts/material (Section 3.1.5) and the coordinate measuring machines (CMMs), which can be either fixed or portable (Section 3.1.6).
  - Destructive scanners. Scanners of this type produce volumetric data by successively removing thin layers of material from the object of study. Examples of scanners of this type are given in Section 3.1.7.

#### 3.1.1. Laser Scanners

Laser scanners collect data that allow the extraction of information about the position of discrete points in space, producing a dense point cloud, which consists of the 3D coordinates of points on the surface of an object of interest. Since the laser scanner also collects color information via a camera sensor, or calculates the intensity of the reflected signal, the point cloud is accompanied by color values (usually RGB) or grayscale values, respectively. The basic function of a laser scanner is the calculation of the distance between the scanning device and discrete points in the surrounding space using laser beams. A transmitter emits and sends a huge number of photons to the object. These photons are reflected on the surface of the object, and a percentage of them ends up in the photosensitive sensor of the laser scanner [51]. The microprocessor of the scanner takes a series of measurements and calculates the distance between the scanning device and the object. The calculation of this distance is accomplished by a triangulation method, or by measuring the time of flight of the pulse, or by measuring the phase difference. Laser scanners may be distinguished into four basic categories, as described in the following [52,53,54].

- Triangulation scanners. They send two laser beams, which intersect at the object of interest. The rays can come either from different sources or from the same source, through splitting the original ray. Triangulation scanners using a single-camera setup include a mechanical base, to the ends of which the following are attached, with known geometry: (a) a transmitter, which sends a laser beam at a defined angle to the object of interest and (b) a camera, which locates the point of intersection of the beam with the object of interest (spot of the laser beam). The transmitter, the camera, and the spot of the laser beam form a triangle, from which the 3D information of the position of the reflection point is derived. The emission angle changes with a predetermined angular step. Typically, the object is scanned via a single scan line to speed up the process. There is also a dual-camera setup with slight variations.
- Time-of-flight scanners. They measure the time required for the laser beam to travel the distance between the emitter and the target and return to the emitter and calculate the distance (*d*) between the emitter and the target based on this time (*t*), given the speed (*u*) of electromagnetic radiation (d=u·t/2). Thus, errors in distance calculation depend on the accuracy of time measurement. They are relatively slow scanners with a range of hundreds of meters or a few kilometers.
- Phase-shift scanners. They use a continuous laser beam instead of discrete pulses. The emitted laser beam hits the target and a part of it is reflected and follows the same path as the emission path, returning to the receiver. They measure the phase shift between the sent and the received waveforms. Phase-shift scanners are fast but correspond to a limited range. Due to the limited possibilities of emitting strong continuous laser radiation, they are used almost exclusively in terrestrial applications (distances up to 100 m).
- Structured light scanners. They use a technology similar to the triangulation method. They project a pattern onto an object with the help of laser beams and study the deformations caused by the object shape, using a camera (or cameras). An important advantage of these scanners is the speed and the consequent ability to calculate the 3D position in space of many points at a time and not of just one point.

#### 3.1.2. Non-Optical Scanners

In the category of non-optical scanners, sonar systems and radar systems basically belong. Sonar (sound navigation and ranging) systems are electro-acoustic devices that use the propagation of sound waves, usually in water (underwater). The acoustic frequencies used in sonar systems vary, from very low (infrasounds) to extremely high (ultrasounds). There are two categories of sonar systems: active and passive. Active sonar scanners have a transmitter and a receiver. They emit an audio signal (i.e., a pulse of sound) and measure the time it takes for the echo of the pulse to return to the receiver (time delay). Passive sonar systems have only a receiver and are able to “hear” sound, without being able to emit sound [55,56].

Radar (radio detection and ranging) systems use radio waves to measure distances. They have a transmitter, which produces electromagnetic waves (radio waves), a transmitting antenna, which emits the pulses of electromagnetic radiation, a receiving antenna (which is usually the same antenna), which receives the reflected radiation, and a receiver, which processes the signal it receives. Their basic operating principle is based on the emission of an electromagnetic pulse at a certain angle to the target and the reception of the reflected radiation. The time between the emission of the pulse and its return is measured by the radar system, and the distance from the object is calculated [57]. There are two types of radar systems:

- Real aperture radar systems, in which the real size of the antenna is considered as its physical size, and
- Synthetic aperture radar (SAR) systems, in which a technique is used to increase the physical length of the antenna using the motion of the flying platform (airborne/satellite) on which the SAR system is installed, and multiple pulse returns are obtained for the same targets, producing images of higher resolution. A frequent use of SAR systems is the production of a digital terrain model through the technique of interferometry, the application of which requires at least two SAR images depicting the same scene, taken either at different times or from a different position [58].

#### 3.1.3. Computed Tomography Scanners

Computed tomography is a digital imaging method, which allows the creation of sections of varying thickness of the structures under examination using an X-ray beam. Two basic types of computed tomography scanners are in use nowadays: fan-beam computed tomography scanners, which are also referred to as computed tomography (CT) scanners, and cone-beam computed tomography (CBCT) scanners.

CT scanners include three main parts [59]: (a) the scanning system, (b) the calculation system, and (c) the image recording and display system. The scanning system includes the X-ray lamp, the array of radiation detectors, and the tomograph table. The X-ray lamp, i.e., the source, is rotated inside a ring, or helically/spirally around the target (part of a patient’s body or object of interest) and emits a fan-shaped beam of X-rays. The latter pass through the target and the signal is weakened by scattering and absorption. The rays leaving the target are collected by radiation detectors. The table where the target is placed has its axis perpendicular to the plane of the detectors and has the ability to move along its axis [60,61]. A combination of two movements is made for scanning the target: a rotational movement (of the lamp around the target) and a linear movement (of the tomograph table). The scanning geometries used in modern fan-beam CT scanners can be distinguished into two main categories: (a) sequential scanning (or scan-move-scan or step and shoot or scan and step) and (b) helical/spiral scanning. In sequential scanning, the lamp is rotated around the target and data are acquired for a single slice. After the lamp is fully rotated, the tomograph table is moved to the next position and the process is repeated. This scanning geometry implies a long data acquisition time and the position and thickness of the sections are determined at the time of scanning and cannot be changed afterwards. In the context of helical/spiral scanning, the X-ray lamp is rotated helically/spirally around the target, while the latter moves continuously along the CT scanner. This synchronous movement of the X-ray lamp and the table implies continuous data acquisition. The calculation system records the attenuation characteristics of the X-ray beam as it passes through the target. The measurements of the weakened radiation recorded by the detectors are converted into electrical signal, which is fed to the computer of the CT scanner. The main problem faced by CT is the calculation of the linear attenuation coefficient at each voxel of each slice and the conversion of the calculated attenuation coefficients to the so-called Hounsfield units or CT numbers [62]. The CT image recording and display system converts for each voxel the Hounsfield units into grayscale values to produce the tomographic image.

CBCT uses a rotating lamp that produces a cone-shaped beam of rays. The lamp is permanently connected antidiametrically to the radiation detector system. The lamp–detector system usually rotates 180°–360° around the target, taking successive tomographic images of the volume of interest [63,64]. CBCT scanners display the tomographic images that they reconstruct from the original structural images in the axial, frontal, and sagittal planes (secondarily reconstructed images). However, in addition to the three basic levels, CBCT scanners may reconstruct images in other levels as well. CBCT delivers much lower doses of radiation to patients than conventional CT [65,66].

#### 3.1.4. Magnetic Resonance Imaging Scanners

Magnetic resonance imaging (MRI) is a diagnostic scanning technique that is based on the principles of magnetic resonance and uses a strong magnetic field of waves and radio frequencies without exposure to radiation. An MRI scanner has a very strong magnet and emits and receives radio frequency waves that interact with hydrogen atoms present in the human body. MRI provides a real-time 3D representation of the tissues and organs of the patient’s body being scanned, with very good soft tissue contrast. The scan takes several minutes and is characterized by increased noise, disadvantages not found in modern CT scanners. The combination of MRI slices leads to the production of a 3D model of the part of the patient’s body being scanned [67].

MRI scanners can be divided into two main categories: closed MRI scanners and open MRI scanners. The main difference between these two types of MRI scanners lies in the location of the magnets. In closed-type MRI scanners, the magnets surround the patient, creating a stronger magnetic field. In open-type MRI scanners, the magnets are placed on top and bottom, and the resulting tomographic images are of lower resolution; however, they do not cause the feeling of claustrophobia in patients, which can be caused in the case of closed-type MRI scanners.

In addition to the usual applications in medicine, MRI scanners are also used for industrial applications.

#### 3.1.5. Ultrasound Scanners

Ultrasound scanners are used for both medical purposes and for industrial applications. Medical ultrasound is an imaging technology that produces images of parts of a body using ultrasound. Ultrasound scanners are devices that generate and send ultrasound to a patient’s body, receive their reflections, process them, and convert them into color or grayscale images. They base their operation on the fact that each tissue of the human body shows a different behavior to ultrasound, and thus reflects, refracts, or absorbs a different percentage of the waves compared to those it receives. The computer system of an ultrasound scanner assigns a color or grayscale value to each tissue, based on the wave reflections, producing the ultrasound images [68]. While conventional 2D ultrasound scanners produce 2D images of the region of interest, 3D ultrasound scanners produce real-time, or near real-time, 3D representations of the volume of interest. The ultrasound equipment consists of a transducer, a scanner, a computer, and a monitor. The transducer is the part of the ultrasound device that comes into direct contact with the patient’s body. It produces the ultrasound pulses when electrical pulses are applied to it, and picks up the echo of the pulses returning to the surface of the body and converts it back into electrical pulses, which are then processed by the computer system of the ultrasound scanner so that they are converted into an image [69]. 3D ultrasound scanners can be distinguished into four main categories, depending on the technique used to acquire the data [70,71], as described below.

- Scanners with a 2D array of transducers. Ultrasound scanners of this type produce an acoustic beam in two dimensions to obtain volumetric data through scanning. The elements of the 2D array of transducers produce a divergent beam in a pyramidal shape, and the received echo is processed to produce a 3D representation.
- Ultrasound scanners with mechanical 3D probes. They have a linear array of transducers within a hand-held instrument. The linear array of transducers can be rotated, tilted, or translated within the probe in a motorized way, under computer control. Thus, the motion mechanisms of the transducer array can be divided into three categories: linear motion, tilt motion, and rotation. In the linear motion of the transducer array, parallel 2D images are acquired for 3D reconstruction. In tilt motion, the transducer takes different tilts to capture the images, with a tilt axis on the surface of the transducer array. In rotational motion, a mechanism rotates the transducer around the central axis of the probe.
- Ultrasound scanners with mechanical localizers. As in ultrasound scanners with mechanical 3D probes, in ultrasound scanners with mechanical locators the latter are motorized. However, while, in scanners with mechanical 3D probes, the scanning mechanism is built into a handheld instrument along with a dedicated 1D linear transducer, a mechanical localizer consists of an external component that holds a conventional 1D transducer to capture a series of consecutive 2D images. The scan path is predetermined so that the relative positions and orientations of the 2D ultrasound images are accurately recorded by the computer system, allowing real-time 3D reconstruction. Motion mechanisms can be separated, as in the case of ultrasound scanners with mechanical 3D probes, into three categories: linear motion, tilt motion, and rotational motion.
- Freehand ultrasound scanners. Ultrasound scanners of this type allow the area of interest to be scanned in different directions and positions, allowing the operator to select the optimal positions and orientations for obtaining the ultrasound images by manually tilting and moving the transducer. The orientation of the transducer is recorded for each tomographic image.

As far as industrial contact ultrasound scanners are concerned, they are mainly used for non-destructive testing purposes to inspect objects (parts, machines, vehicles, etc.). Ultrasonic inspection uses sound waves with frequencies above the audible range. Most testing is accomplished in the 1–5 MHz range, but in specialized applications frequencies in the 20 kHz–100 MHz range are used. When ultrasound scanners are used for inspection purposes, defects in the test objects are detected if a change in acoustic impedance is observed along the path of the ultrasound beam. For instance, an open-air crack has very low acoustic impedance and, thus, reflects almost all the acoustic energy applied to it. Thus, cracks can be detected, for example, due to an increase in the reflected signal. The time of arrival of the reflected signal at the ultrasound scanner reproduces information about the crack location, since the speed of sound in the material is known [72]. Contact ultrasound scanners are used for inspection of various types of objects, components, machinery, and vehicles and produce both 3D and 2D images for aerospace, petrochemical, electric power, automotive, and others related industries [73,74]. In addition to the contact ultrasonic scanners used in industrial applications, with transducers that come into contact with the study object, there are also non-contact ultrasound scanners, as mentioned in Section 3.1.2 (subcategory of the non-optical non-contact sonar systems, which do not touch the test material).

#### 3.1.6. Coordinate Measuring Machines

Coordinate measuring machines (CMMs) are non-destructive testing (NDT) scanners, as they do not cause any change/damage to the object they scan and are mainly used for industrial applications. They collect a set of points in space for a specific object, with the help of a sensor, describing its 3D geometry. Their basic principle lies in the determination of the position of the sensor by calculating its displacement relative to a reference position along the X, Y, and Z axes of a 3D Cartesian coordinate system. In addition to the sensor displacement along the X, Y, and Z axes, many CMMs allow control of the tilt angle of the sensor. Scanning via CMMs may be carried out either in an operator-controlled manner or by a computer. CMMs include three main parts: (a) the main structure, which includes the three axes of movement, (b) the system of the contact sensor, and (c) the data collection and processing system. They are divided into two main categories: fixed CMMs and portable CMMs.

Fixed CMMs are further distinguished into four basic types, as described below [75,76,77].

- Bridge-type CMMs. This is the most common type of CMM. A bridge, on which the Z-axis lies, moves on the base of the machine. The measuring head is located on the Z-axis and can be moved along this axis (up and down), along the X-axis (i.e., the axis along the bridge), and along the Y-axis (perpendicular to the axis of bridge) by moving the entire bridge over the CMM base.
- Cantilever-type CMMs. In this kind of CMM, the measuring head is attached to one side of a rigid base. They are used for measuring smaller objects (e.g., parts of objects) than those measured by bridge-type CMMs. They provide a high level of accuracy.
- Horizontal arm CMMs. They provide lower accuracy of measurements than bridge-type CMMs and cantilever-type CMMs. They are particularly useful for measurements of larger objects and objects that involve measurements in hard-to-reach places (e.g., for automotive use, to scan cars and their internal parts).
- Gantry CMMs. The structure of this kind of CMM is similar to the structure of bridge-type CMMs, but they are much larger than the latter. The bridge is raised on pillars. They provide a high level of accuracy and are used for measuring large volume objects (e.g., for use in the aeronautical industry).

Portable CMMs are basically articulating arms. They are moved to the point of interest, being suitable for scanning objects with dimensions up to 10 m.

#### 3.1.7. Destructive Scanners

Destructive contact scanners produce volumetric data by successively removing thin layers of material. The generated slice images are combined together to represent the 3D volume of the object of interest. Examples of scanners of this type are listed below.

- Serial block-face scanning electron microscopy (SBEM) scanners. Instruments of this type use a microtome (i.e., a special cutting device that produces extremely thin sections) inside a scanning electron microscope, i.e., a microscope used to examine the microstructure of objects that uses a high-energy electron beam to create an image of the study object on a computer screen. The microtome cuts the object of interest, and, through the microscope, the sections are visible. The process is repeated until the entire object is digitized, and thus completely destroyed. Scanners of this type provide precision of the order of a few nanometers [78].
- Knife-edge scanning microscopy (KESM) scanners. Instruments of this type combine the sectioning of the study object and the visualization of the section in one step. They use an arm with a diamond knife to cut the object [79].
- Micro-optical serial tomography (MOST) scanners. Instruments of this type consist of a microtome, a light microscope, and an image recorder, and perform imaging and sectioning simultaneously [80].
- Focused-ion-beam scanning electron microscopy (FIBSEM) scanners. In instruments of this type, a scanning electron microscope equipped with a focused beam of gallium ions is used. The gallium ions gradually impinge on the object of interest, causing the surface atoms of the object to be ejected and its surface to become amorphous. The detector of the backscattered electrons of the instrument is used for image surfaces, creating a large series of images that can be combined for 3D representation of the object of interest [81].

### 3.2. From Scans to 3D Models

Scanners generally produce point clouds or volumetric representations in the form of tomographic images (slices). In this section, an overview of conversion of scans to 3D mesh models is given.

#### 3.2.1. Point Clouds to 3D Models

The point clouds derived from laser scanners are stored in a local coordinate system (scanner system). Therefore, if an area has been scanned from two or more positions, a 3D Euclidean transformation between the point clouds has to be computed in order to transform them into a single coordinate system before merging the point clouds. Usually, special retroreflective targets are placed in the area being scanned, which are automatically recognized by the software accompanying the scanner. In the case where at least three targets have been measured by topographical methods in a ground reference system, the point clouds obtained by the scanner can be transformed from the local reference system of the scanner to the ground reference system, and then the point clouds can be merged. In the case of unavailability of topographic measurements, the point clouds can be transformed into a common reference system using at least three common points, considering one of the individual scans as the reference scan, and then the clouds can be merged.

However, the existence of retroreflective targets is not a prerequisite for the transformation of the different scans into a single system. Cloud merging may be performed based on overlaps in successive scans, using homologous points, to calculate the transformation. A manual process of selection of such points and matching them may be implemented, or, usually, an automatic process of merging the point clouds based on common points may be performed. The last category of methods includes the frequently used ICP (iterative closest point) point cloud merging method, which requires the existence of a large percentage of overlap of the clouds and the availability of an initial estimation of the transformation parameters [82].

Whether the point cloud has come from merging of different scans or from a single scan, it usually requires further processing, which may include outlier removal, denoising, etc. If the application requires the creation of a 3D surface, it can be created using the processed point cloud. Finally, if the scanning device also includes a camera that captures images of the target, or if the latter is photographed via a different camera, a texture mapping process can be applied to the 3D model. In this case, the interior and exterior orientation parameters of the images have to be calculated, usually through an SfM-MVS pipeline (Section 2.1).

#### 3.2.2. Tomographic Images to 3D Models

The 3D reconstruction of the region of interest depicted in tomographic images captured by a scanner (e.g., CT/CBCT/MRI/ultrasound scanner) is based on image segmentation, i.e., on dividing each tomographic image into individual non-overlapping distinct regions (segments) homogeneous with respect to some feature of the image, e.g., brightness or texture [83]. Segmentation may be performed both manually and automatically. Segmentation methods can be distinguished into two main categories:

- Intensity-based methods, such as thresholding, edge detection, and active contours.
- Geometry-based methods, such as region growing and clustering.

Thresholding is based on the existence of two classes of pixels with different intensity values and requires the definition of an appropriate pixel intensity threshold value or a range of intensity values, within which the pixels of the object of interest are considered to belong. Pixels with intensity greater or less than the specified value or those belonging to the specified value range are considered to belong to the object of interest and the corresponding areas are included in the reconstructed volume. The remaining volumes are not part of the reconstruction. One of the main disadvantages of thresholding is the fact that the tomographic images are often characterized by uneven illumination and/or noise, and thus the method may not yield satisfactory results in the detection of the regions of interest. However, these problems may be corrected by specific techniques, e.g., use of noise reduction filters, etc.

As far as edge detection is concerned, edges, in an image, are linear elements that constitute boundaries between areas of different intensity, that is, they are lines on either side of which a significant change in image intensity is observed. At the edges, the first oriented derivative of the image intensity function is maximized, i.e., the gradient becomes maximal. Therefore, they can be detected based on this property and a threshold, above which a point can be considered as an edge point. In addition, edge detection can be performed using the second derivative, by calculating the Laplacian as the second derivative, representing the change in gradient, which zeroes out on the edge, presenting a local maximum and minimum on either side of it. Moreover, many edge detection techniques have been developed, e.g., the Canny edge detector [84]. Often, before applying any edge detection method to tomographic images, noise reduction is required, which can be achieved in various ways, e.g., by filtering.

Active contour methods use closed deformable curves, which evolve (move) dynamically from an initial position towards the boundaries of the imaged objects. Two types of active contour methods can be distinguished: parametric and geometric. In parametric methods, contours are in the form of 2D parametric curves. In geometric methods, contours are defined with the help of level sets and the evolution of the curve is independent of its parameterization. Both categories of methods are based on the minimization of an energy function [85].

Region growing consists of finding areas of the images based on their geometric characteristics [86]. The points of the regions are connected to each other based on predefined criteria. These criteria can be determined based on pixel intensity values or image edges, in which case the region development method is combined with an edge detection technique. In its simplest version, a region growing method requires the manual definition of an initial point or some initial points (seed points) belonging to different regions of the image. This procedure can be performed on one or more tomographic images. Then, each region defined by one or more seed points is expanded by adding neighboring pixels, i.e., by locating all pixels connected to these seed points based on the defined criteria. An example of a possible criterion concerns the growth of the region until it meets an edge of the image [87]. The Watershed method is a frequently used region growing method. It uses concepts from edge detection to divide images into homogeneous regions. However, it can cause the image to be fragmented into an unreasonably large number of segments [88].

After the implementation of a segmentation process to the whole set of tomographic images, the segments are automatically converted into 3D mesh models, which may be further processed, usually by converting them into point clouds and implementing cloud cleaning techniques, before converting them back to 3D meshes and applying further processing.

### 3.3. Discussion

Nowadays, there is great discussion on which technology should be used for acquiring 3D reconstructions of different kinds of targets. The answer to this question lies in the object/scene/human body to be reconstructed. For common topographic applications, both image-based 3D modeling and scanner-based 3D modeling (more commonly, via a laser scanner) may be applied, or a combination of these sensors may be the ideal solution, with the choice depending on several factors, such as cost, availability of equipment, size of object, and other object characteristics. In other applications, the choice of a specific type of scanner is obligatory, depending on the type of the object to be modeled, e.g., MRI scans are better suited for soft tissue reconstruction compared to CT/CBCT scans in medical applications, fetuses of pregnant women should be modeled using ultrasound scanners rather than using CT/CBCT scanners, objects of large volume should be modeled through gantry CMMs rather than cantilever-type CMMs or portable CMMs, etc. The characteristics of the different types of common scanners presented in Section 3.1 of this article would be useful in determining the right sensor for specific 3D modeling applications.

## 4. Applications

In addition to the classic topographic applications of 3D modeling of sites/objects (such as, for example, geometric documentation of monuments and cultural heritage sites, production of 3D models of cities and buildings, 3D modeling of small objects/relics, etc.) the creation of 3D models using scanners is required for a variety of special applications. Indicative specialized applications of 3D modeling are presented in this section.

### 4.1. Medical and Dental Applications

3D models of organs/tissues of the human body or fetuses of pregnant women are produced using multiple 2D tomographic images, e.g., from CT/CBCT/MRI/ultrasound scanners. These 3D models can either be printed by a 3D printer or viewed on a computer screen, allowing additional measurements to be taken on them. For example, printed or digital 3D models of human organs are useful in surgery, for diagnostic purposes, and/or for surgery/treatment planning [89,90,91,92]. Moreover, they can be used for surgeon training purposes, through realistic simulation of surgical procedures [93]. Another widespread use of 3D modeling and 3D printing in medical and dental applications is the production of personalized implants adapted to the patient [94] and the production of 3D printed prosthetic limbs, as their 3D design, when it is personalized, is based on the 3D anatomy of the specific part of a patient’s body, acquired by medical scanners [94,95,96,97]. Efforts have also been made to 3D design artificial organs and tissues based on data from medical scanners [98].

### 4.2. Applications in the Computer Graphics Industry

3D models derived from scanners have been used for many years in the film industry and video game development industry, as well as in virtual and augmented reality applications, as they are easier to be created using scanners in comparison to their 3D design from scratch. The 3D models may belong to either objects or sites, even to humans, involving 3D modeling of both their faces and their whole bodies [99,100,101].

### 4.3. Cultural Heritage Applications in the Field of Culture

In addition to the conventional applications of 3D modeling in the field of culture (3D modeling and geometric documentation of cultural heritage sites/buildings/monuments and 3D modeling of objects/relics of high cultural value), in recent years, emphasis has also been placed on 3D modeling of intangible cultural heritage [102]. Such examples concern the 3D modeling of dances through the 3D recording of dancers’ dance movements [103,104], the 3D recording of athletes’ movements, in some cases in combination with the surrounding environment and/or objects [105], and the 3D motion recording for traditional crafts [106,107]. In addition, 3D modeling applications of underwater spaces or cultural heritage objects are of special interest [108].

### 4.4. Applications in the Fields of Safety and Rescue

Rapid or real-time 3D modeling of areas affected by a natural disaster (e.g., earthquake) or man-made disaster (e.g., terrorist attack) has significant use in the field of search and rescue. Research has been conducted on the use of such 3D models by search and rescue teams to quickly make decisions and determine the location of trapped people or people in need of help [109,110,111]. Moreover, the creation of 3D models for places where an accident or criminal act occurred, as well as for places affected by a natural disaster, contributes to the subsequent analysis of the event and the drawing of conclusions about it [112,113,114].

### 4.5. Reverse Engineering Applications in the Manufacturing Industry

Reverse engineering can be defined as the process of extracting design information from an existing product. Unlike the traditional manufacturing process, whereby a product, or part of it, is firstly digitally designed and then manufactured, a reverse engineering process starts from a real object and attempts to create its 3D geometric model. It is based on the assumption that an existing object embodies the main specifications of the product to be designed. 3D modeling for reverse engineering purposes has been used in the manufacturing industry for the purposes listed below [115].

- New product design. In some new product design applications, the design starts from a physical (existing) prototype object. Especially for objects with freeform surfaces, it is easier to produce their 3D polygonal model through a reverse engineering process and post-build the CAD (computer-aided design) model based on the polygonal model.
- Modification of an existing product. Existing product designs are often iteratively modified. However, the CAD 3D model for a product after modification may not be available, and its 3D model may have to be created from scratch through a reverse engineering technique.
- Loss of digital 3D product designs. In some cases, the 3D model of a product, or part of it, is no longer available or has been destroyed (e.g., car/aircraft/ship parts that have been retired).
- Product verification. In some applications it is useful to generate the 3D model of a product using overlapping images or scans of it, or part of it, and subsequently compare it with the 3D CAD model of its design to identify any deviations.
- Quality control and inspection. 3D modeling of parts/machines/vehicles and other products using scanners is conducted to detect cracks or other types of defects in the object under consideration for quality control.

At this point, it should be mentioned that, for reverse engineering applications related to the creation of product designs for production purposes, the 3D polygonal models resulting from scanners need to be converted into CAD models using a related software.

## 5. Conclusions

Advances in the scientific fields of photogrammetry and computer vision have led to the development of automated multi-image methods that solve the problem of 3D reconstruction. Simultaneously, 3D scanners have become a common source of acquisition of data required for 3D modeling of real objects/scenes/human bodies. This article presents a comprehensive overview of different 3D modeling technologies that may be used to reconstruct the outer or inner surfaces of different kinds of targets. Hence, it covers the topics of 3D modeling using images and scanners of different categories, the basic principles of which are also outlined. Finally, it discusses, indicatively, some non-standard applications of 3D modeling, except for well-established conventional ones.

## Figures and Tables

**Table 1:** 3D modeling methods discussed in this review article.

Data	Methods
Images	Multi-view stereo
	Two-image reconstruction
	Conventional photogrammetric procedure
	Shading-based shape recovery
	Usage of a stereo-camera
	Usage of satellite imagery
Scans	Point clouds to 3D models
	Tomographic images to 3D models

## Data Availability

Not applicable.
